# Decreased Reactive Oxygen Species Signaling Alters Glutamate Receptor Transport to Synapses in *C. elegans* AVA Neurons

**DOI:** 10.17912/micropub.biology.000528

**Published:** 2022-03-17

**Authors:** Rachel L Doser, Frederic J Hoerndli

**Affiliations:** 1 Department of Biomedical Science, Colorado State University, Fort Collins, Colorado 80523

## Abstract

Reactive oxygen species (ROS) are chemically reactive molecules normally produced during cellular respiration. High ROS levels negatively impact forms of synaptic plasticity that rely on changes in the number of ionotropic glutamate receptors (iGluRs) at synapses. More recently, we have shown that physiological increases in ROS reduce iGluR transport to synapses by acting on activity-dependent calcium signaling. Here, we show that decreasing mitochondria-derived ROS decrease iGluR transport albeit in a calcium-independent manner. These data demonstrate differential regulatory mechanisms by elevated or diminished ROS levels which further support a physiological signaling role for ROS in regulating iGluR transport to synapses.

**Figure 1. Diminished ROS levels decrease GLR-1 transport out of the cell body via a calcium-independent mechanism.**
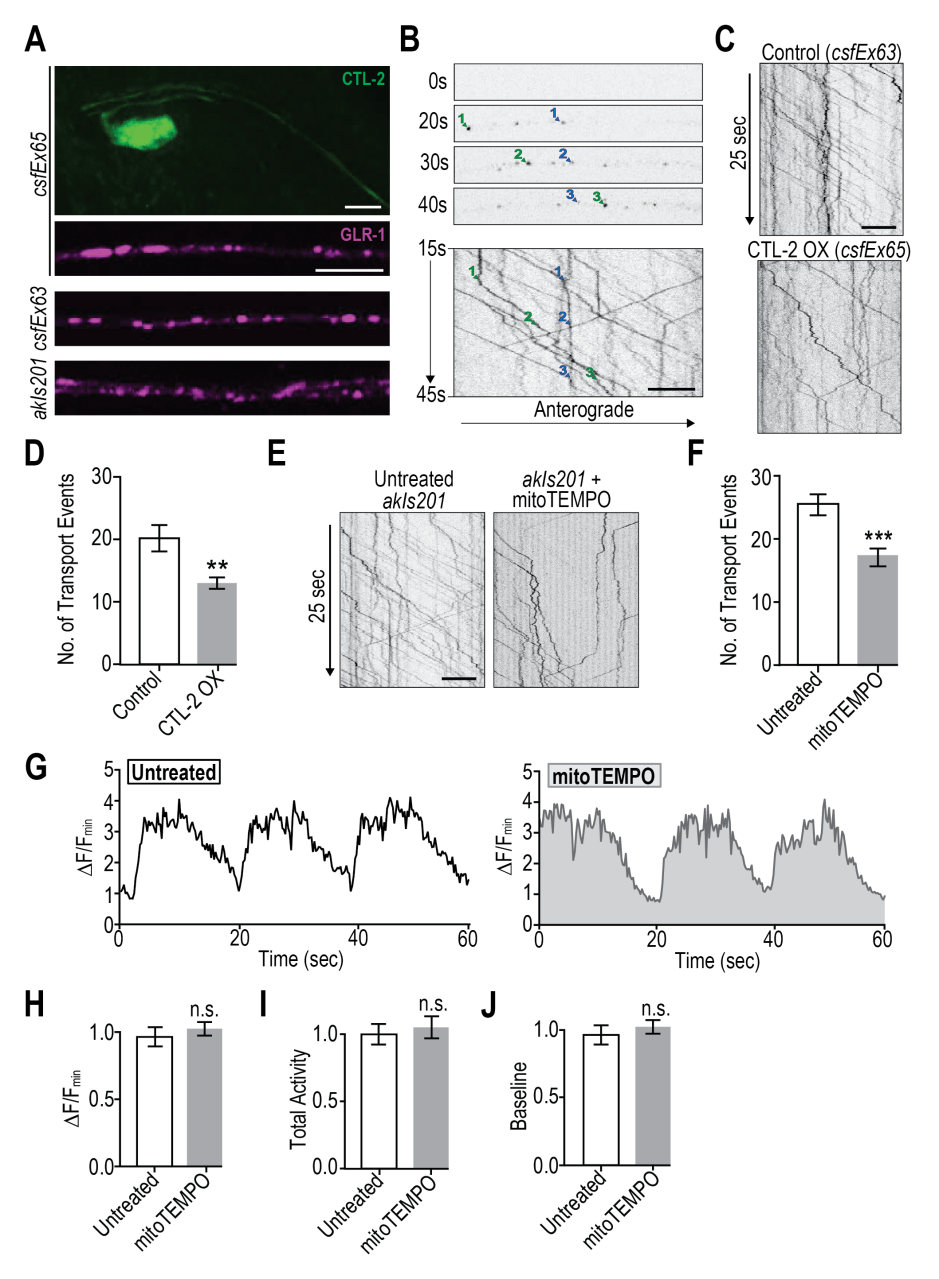
A) Representative images showing expression of contents of transgenic arrays used. Scale bar = 5 µm. B) GLR-1::mCherry transport. Top: Single time points from 50 s of continuous imaging (100 ms/frame). Two GLR-1-containing vesicles (blue and green numbers) are pointed out for three timepoints. Bottom: Kymograph derived from 30 s of a 50 s image stream depicting position (x-axis) of GLR-1 transport vesicles over time (y-axis). Scale bar = 5 µm. C and E) Representative kymographs depicting GLR-1 transport vesicles (black lines) as they are transported through the length of the neurite (x-axis) over time (y-axis). Scale bar = 5 µm. D) Quantification of transport events in control ( *lin-15(n765ts)* X; *glr-1(ky176)* III; *csfEx63* , n=15) and worms overexpressing catalase (CTL-2(OX)) in the AVA ( *lin-15(n765ts)* X; *glr-1(ky176)* III; *csfEx65* , n=11, **:p=0024, two-tailed Student’s t-test). F) Quantification of transport events from untreated (n=18) and mitoTEMPO treated (n=20) worms ( *glr-1(ky176)* III; *akIs201* , ***:p=0.0008, two-tailed Student’s t-test). G) Representative traces of changes in GCaMP6f fluorescence in the AVA cell body over 60 s *in vivo* normalized to baseline fluorescence (DF/F _min_ ) from untreated (n=35) and mitoTEMPO treated (n=35) worms ( *lin-15(n765ts) X; csfEx62* ). H) Average DF/F _min_ normalized to untreated controls. I) Total activity (sum of fluorescence values above baseline divided by baseline) normalized to untreated controls. J) Average baseline of these groups normalized to untreated controls.

## Description

In the brain, signal transmission between neurons mainly occurs at electrochemical junctions or synapses where release of presynaptic neurotransmitters activates postsynaptic receptors. In most nervous systems, the primary excitatory neurotransmitter is glutamate. When released by presynaptic neurons, it activates glutamate receptors containing cation channels and causes excitation through membrane depolarization. The AMPA subtype (AMPAR) of glutamate receptors is especially central to excitatory transmission (Ashby et al., 2008). The amplitude of a postsynaptic response to glutamate release depends on the number of postsynaptic AMPARs, and changes in synaptic content of AMPARs is the basis for the synaptic plasticity that underlies learning and memory (Groc and Choquet, 2020). Most AMPARs are synthesized in the cell body and must undergo long-distance transport to these sites. This transport is a multistep process involving loading of AMPAR-containing vesicles onto molecular motors (Esteves da Silva et al., 2015; Hangen et al., 2018; Hoerndli et al., 2013; Kim and Lisman, 2001; Setou et al., 2002), delivery of these vesicles (Heisler et al., 2014; Setou et al., 2002), and exocytosis of AMPARs to the synaptic membrane (Yudowski et al., 2007). Regulation of receptor endocytosis (Ehlers, 1999; Sathler et al., 2021) and surface diffusion (Choquet and Triller, 2013) further contribute to controlling the synaptic content of AMPARs.

Regulation of motor-dependent transport and delivery are the least understood steps, but we now know they are regulated by activity-dependent calcium signaling (Hangen et al., 2018; Hoerndli et al., 2015). Additionally, we have recently shown in *C. elegans* that the calcium influx that upregulates AMPAR transport and delivery is attenuated by increased reactive oxygen species (ROS; Doser et al., 2020), a class of reactive molecules that are normal byproducts of oxidative phosphorylation (Halliwell, 1992). These findings begin to explain observations of synaptic plasticity defects (i.e. less induction) in elevated ROS conditions (Bliss and Collingridge, 1993; Kamsler and Segal, 2003; Klann, 1998). Interestingly, ROS depletion leads to similar defects in synaptic plasticity (Gahtan et al., 1998; Kishida and Klann, 2006), suggesting that ROS concentrations must be within a specific physiological range for normal synaptic plasticity. So, we asked if ROS are required for AMPAR transport by depleting ROS via pharmacological and genetic methods in *C. elegans* then analyzing transport of the AMPAR subunit GLR-1 (Doser et al., 2020).

In *C. elegans* , the AVA glutamatergic interneurons are ideal for these studies because they are unipolar with a single neurite that spans the length of the entire ventral nerve chord (Maricq et al., 1995). Additionally, cell-specific promoters for the AVA neuron allow for molecular replacement of native GLR-1 with a fluorescent-tagged GLR-1 in these neurons alone (Figure 1 A). This has enabled visualization of the transport of individual vesicles containing GLR-1 as they are exported from the AVA cell body through the long AVA neurite (Doser et al., 2020; Hoerndli et al., 2015). Using these methods, we have shown decreased GLR-1 transport when ROS levels were elevated within the physiological range (Doser et al., 2020). Since we were able to pinpoint that elevated ROS downregulates GLR-1 transport by attenuating activity-dependent calcium influx, we hypothesized that ROS act as regulatory molecules in this process.

If this hypothesis is correct, then reducing ROS would alter the amount of GLR-1 transport. To test this, we overexpressed the neuronal catalase encoded by the *ctl-2* gene in AVA (Figure 1A, top), which would presumably lead to decreased ROS levels only in these neurons. To image GLR-1 transport, we photobleached a proximal portion of the AVA neurite to uncover the dim fluorescence of GLR-1-containing vesicles (Figure 1 B). Then, we continuously imaged GLR-1::mCherry for 50 s at a single confocal plane within a section of the AVA neurite. Transport events within this image stream are represented in kymographs as black traces with position on the x-axis and time on the y-axis (Figure 1 B, C and E). Quantification of the number of GLR-1 transport events from kymographs revealed that *C. elegans* with AVA-specific overexpression of CTL-2 had decreased transport compared to the control strain (Figure 1 D). It is important to note that this difference in transport between strains is unlikely due to discrepancies in their GLR-1::mCherry expression since the average fluorescence of GLR-1::mCherry in the AVA neurite is nearly the same in each ( *csfEx63* : 470.4 ± 66.1 and *csfEx65* : 465.6 ± 68.2; mean ± SEM; p = 0.95, two-tailed Student’s t-test). These results suggest that chronic CTL-2 overexpression decreased ROS levels which in turn led to a reduction in GLR-1 transport. A major source of ROS is mitochondrial oxidative phosphorylation (Halliwell, 1992), so we hypothesized that CTL-2 overexpression decreased the effect of mitochondrial-derived ROS signaling.

To test this hypothesis and rule out developmental effects of CTL-2 overexpression, we treated *C. elegans* with mitoTEMPO, a mitochondrial-targeted antioxidant (Murphy and Smith, 2007), to acutely diminish ROS signaling. Following a 2-hour mitoTEMPO treatment, we found that GLR-1 transport was decreased compared to untreated controls (Figure 1 E and F). The similar decrease in transport due to CTL-2 overexpression and acute mitoTEMPO treatment suggests two things: First, diminished ROS signaling has a direct effect on the regulation of GLR-1 transport. Second, since mitochondria-targeted antioxidants reduced transport to the same extent as overexpression of a cytoplasmic catalase, it is likely that mitochondrial respiration is a major contributor to ROS signaling involved in regulation of GLR-1 transport.

Since we previously determined that elevated ROS regulates GLR-1 transport by attenuating calcium influx (Doser et al., 2020), we next asked whether diminished ROS levels also attenuate calcium influx. To address this question, we subjected a *C. elegans* strain expressing the calcium indicator GCaMP6f in AVA neurons to a 2-hour mitoTEMPO treatment. Quantification of GCaMP fluorescence over a 60 s imaging session (Figure 1 G) did not reveal any changes in the amplitude of GCaMP peaks (Figure 1 H), total activity (summation of GCaMP fluorescence above baseline, Figure 1 I), or baseline GCaMP fluorescence (Figure 1 J) between untreated and mitoTEMPO treated worms.

Altogether, these results demonstrate that diminished ROS levels decrease the amount of GLR-1 transport out of the cell body via a mechanism that seems to be independent of activity-dependent calcium influx based on *in vivo* calcium imaging with GCaMP6f. However, since calcium imaging is not as sensitive as other measures of calcium influx, it remains possible that calcium channel activation, conductance, or inactivation is altered by diminished ROS signaling. The addition of these results to our previous findings (Doser et al., 2020) suggest that ROS are a necessary regulator of long-distance AMPAR transport. Since this transport is crucial for supplying receptors in a way that allows for synaptic plasticity, these studies begin to explain why synaptic plasticity defects are observed in conditions of non-physiological ROS.

## Methods

CLONING.

The catalase gene ( *ctl-2)* was cloned from *C. elegans* cDNA using the forward primer 5’-GGGGACAAGTTTGTACAAAAAAGCAGGCTATGCCAAACGATCCATCGGA-3’ and reverse primer 5’-GGGGACCACTTTGTACAAGAAAGCTGGGTCGATATGAGAGCGA GCCTGTTTC-3’designed on ApE (v.2.0.60). Using the gateway recombination cloning technique (Invitrogen), the *ctl-2* gene was positioned behind P *flp-18* , an AVA specific promoter, and followed by an eGFP 3’UTR (from pGH112, Erik Jorgensen) in the destination vector pCFJ150 (Erik Jorgensen, Addgene - Plasmid #19329).

TRANSGENIC STRAINS.

Transgenic strains were created by microinjection of *lin-15(n765ts)* worms as previously described (Doser et al., 2020). The above plasmids were used to create the following extrachromosomal arrays: *csfEx65 [Pflp-18::ctl-2::eGFP* + *Prig-3::glr-1* :: *mCherry* ], *csfEx63 [Prig-3* :: *glr-1* : *:mCherry* ] and *csfEx62 [Prig-3::GCaMP6f* + *lin-15p::lin-15* ]. The *csfEx65* and *csfEx63* transgenic strains have a loss-of-function mutation in *glr-1* (allele: *ky176* ) in addition to *lin-15* ( *n765ts).*

IMAGING AND ANALYSIS.

Confocal microscopy was carried out using a spinning disc confocal microscope (Olympus IX83) as previously described (Doser et al., 2020).

*IN VIVO* GLUTAMATE RECEPTOR IMAGING AND ANALYSIS.

*In vivo* imaging of glutamate receptors was conducted on strains containing either extrachromosomal *Prig-3* :: *glr-1* :: *mCherry* ( *csfEx65* and *csfEx63* ) or *Prig-3::SEP::mCherry::glr-1* ( *akIs201* ; Hoerndli et al., 2015) in a *glr-1* null background ( *glr-1(ky176)* ) *.* All imaging experiments were done using one-day-old adults as described in more detail in Doser et al., 2020.

*IN VIVO* CALCIUM IMAGING.

Calcium imaging experiments were conducted on strains containing the extrachromosomal array *csfEx62* in the *lin-15(n765ts)* genetic background. Eight to ten one-day-old adult animals were placed on a 10% agar pad with 2 µL of M9 buffer. They were imaged individually at 10x for 60 seconds (image stream was acquired using the 488nm excitation laser with a 250 ms exposure time for 240 frames). The fluorescence was measured and analyzed as previously described (Doser et al., 2020).

CODE AVAILABILITY.

Custom Excel modules used to analyze *in vivo* calcium imaging can be found at https://github.com/racheldoser/GCaMP_Analysis_Excel_VBA.git .

STATISTICAL ANALYSIS.

All datasets were screened for potential outliers using a Thompson Tau test. Cleaned datasets were then tested for statistical significance using a two-tailed Student’s T-test.

MITOTEMPO TREATMENT.

A stock of 1M mitoTEMPO (Sigma) was double dissolved in deionized water and diluted to 0.5 mM with M9 and OP50 liquid culture immediately before the 2-hour treatment as previously described (Xu and Chisholm, 2014). 20-40 one-day-old adult control worms were individually picked off NGM/OP50 plates and placed into a 1.5 mL Eppendorf tube containing either control media (M9/OP50) or 0.5 mM mitoTEMPO in M9/OP50. The tubes were placed on a rocker to allow for oxygenation during the duration of the treatment. The worms were then pipetted out of the tubes onto fresh NGM/OP50 plates immediately before being moved to an agar pad for imaging as described above.

## Reagents

All reagents created in our lab may be shared upon reasonable request.

**Table:** 

**STRAIN**	**GENOTYPE**	**SOURCE**
FJH 15	*glr-1(ky176) III; akIs201*	Hoerndli Lab, Colorado State University
FJH 289	*lin-15(n765ts) X; glr-1(ky176) III; csfEx65*	Hoerndli Lab, Colorado State University
FJH 188	*lin-15(n765ts) X; glr-1(ky176) III; csfEx63*	Hoerndli Lab, Colorado State University
FJH 186	*lin-15(n765ts) X; csfEx62*	Hoerndli Lab, Colorado State University
**INTEGRATED ARRAYS**	**CONTENTS**	**SOURCE**
*akIs201*	*Prig-3::SEP::mCherry::glr-1*	Hoerndli Lab, Colorado State University
EXTRACHROMOSOMAL ARRAYS	CONTENTS	SOURCE
*csfEx62*	*Prig-3::GCaMP6f + Plin-15::lin-15 + Pegl-20::nls::DsRed*	Hoerndli Lab, Colorado State University
*csfEx63*	*Prig-3::glr-1::mCherry + Plin-15::lin-15*	Hoerndli Lab, Colorado State University
*csfEx65*	*Pflp-18::ctl-2::eGFP::let-858 + Prig-3::glr-1::mCherry + Plin-15::lin-15*	Hoerndli Lab, Colorado State University
**PLASMID NAME**	**GENE/INSERT**	**SOURCE**
pRD21	*Pflp-18::ctl-2::eGFP::let-858*	Hoerndli Lab, Colorado State University
pAS1	*Prig-3::GCaMP6f::unc-54*	Stetak Lab, University of Basel
pDM1556	*Prig-3::glr-1::mCherry*	Maricq Lab, University of Utah
pJM23	*Plin-15::lin-15*	Maricq Lab, University of Utah
pCT61	*Pegl-20::nls::DsRed*	Maricq Lab, University of Utah
**PHARMACOLOGICAL AGENT**	**EFFECT**	**SOURCE**
MitoTEMPO	An antioxidant that accumulates in mitochondria due to conjugation to a lipophilic cation.	Sigma-Aldrich

## References

[R1] Ashby, M., Daw, M., Issac, J., 2008. AMPA Receptors, in: Gereau, R., Swanson, G. (Eds.), The Glutamate Receptors. Humana Press, Totowa, NJ, pp. 1–44.

[R2] Bliss TV, Collingridge GL (1993). A synaptic model of memory: long-term potentiation in the hippocampus.. Nature.

[R3] Choquet D, Triller A (2013). The dynamic synapse.. Neuron.

[R4] Doser RL, Amberg GC, Hoerndli FJ (2020). Reactive Oxygen Species Modulate Activity-Dependent AMPA Receptor Transport in
*C. elegans*
.. J Neurosci.

[R5] Ehlers MD (1999). Synapse structure: glutamate receptors connected by the shanks.. Curr Biol.

[R6] Esteves da Silva M, Adrian M, Schätzle P, Lipka J, Watanabe T, Cho S, Futai K, Wierenga CJ, Kapitein LC, Hoogenraad CC (2015). Positioning of AMPA Receptor-Containing Endosomes Regulates Synapse Architecture.. Cell Rep.

[R7] Gahtan E, Auerbach JM, Groner Y, Segal M (1998). Reversible impairment of long-term potentiation in transgenic Cu/Zn-SOD mice.. Eur J Neurosci.

[R8] Groc L, Choquet D (2020). Linking glutamate receptor movements and synapse function.. Science.

[R9] Halliwell B (1992). Reactive oxygen species and the central nervous system.. J Neurochem.

[R10] Hangen E, Cordelières FP, Petersen JD, Choquet D, Coussen F (2018). Neuronal Activity and Intracellular Calcium Levels Regulate Intracellular Transport of Newly Synthesized AMPAR.. Cell Rep.

[R11] Heisler FF, Lee HK, Gromova KV, Pechmann Y, Schurek B, Ruschkies L, Schroeder M, Schweizer M, Kneussel M (2014). GRIP1 interlinks N-cadherin and AMPA receptors at vesicles to promote combined cargo transport into dendrites.. Proc Natl Acad Sci U S A.

[R12] Hoerndli FJ, Maxfield DA, Brockie PJ, Mellem JE, Jensen E, Wang R, Madsen DM, Maricq AV (2013). Kinesin-1 regulates synaptic strength by mediating the delivery, removal, and redistribution of AMPA receptors.. Neuron.

[R13] Hoerndli FJ, Wang R, Mellem JE, Kallarackal A, Brockie PJ, Thacker C, Madsen DM, Maricq AV (2015). Neuronal Activity and CaMKII Regulate Kinesin-Mediated Transport of Synaptic AMPARs.. Neuron.

[R14] Kamsler A, Segal M (2003). Hydrogen peroxide modulation of synaptic plasticity.. J Neurosci.

[R15] Kim CH, Lisman JE (2001). A labile component of AMPA receptor-mediated synaptic transmission is dependent on microtubule motors, actin, and N-ethylmaleimide-sensitive factor.. J Neurosci.

[R16] Kishida KT, Klann E (2007). Sources and targets of reactive oxygen species in synaptic plasticity and memory.. Antioxid Redox Signal.

[R17] Klann E (1998). Cell-permeable scavengers of superoxide prevent long-term potentiation in hippocampal area CA1.. J Neurophysiol.

[R18] Maricq AV, Peckol E, Driscoll M, Bargmann CI (1995). Mechanosensory signalling in C. elegans mediated by the GLR-1 glutamate receptor.. Nature.

[R19] Murphy MP, Smith RA (2007). Targeting antioxidants to mitochondria by conjugation to lipophilic cations.. Annu Rev Pharmacol Toxicol.

[R20] Sathler MF, Khatri L, Roberts JP, Schmidt IG, Zaytseva A, Kubrusly RCC, Ziff EB, Kim S (2021). Phosphorylation of the AMPA receptor subunit GluA1 regulates clathrin-mediated receptor internalization.. J Cell Sci.

[R21] Setou M, Seog DH, Tanaka Y, Kanai Y, Takei Y, Kawagishi M, Hirokawa N (2002). Glutamate-receptor-interacting protein GRIP1 directly steers kinesin to dendrites.. Nature.

[R22] Xu S, Chisholm AD (2014). C. elegans epidermal wounding induces a mitochondrial ROS burst that promotes wound repair.. Dev Cell.

[R23] Yudowski GA, Puthenveedu MA, Leonoudakis D, Panicker S, Thorn KS, Beattie EC, von Zastrow M (2007). Real-time imaging of discrete exocytic events mediating surface delivery of AMPA receptors.. J Neurosci.

